# Laryngotracheal mold for stenting in pediatric glottic and subglottic stenosis

**DOI:** 10.1017/S0022215125102533

**Published:** 2025-09

**Authors:** Flavia Varela, Kishore Sandu

**Affiliations:** 1 Department of Otorhinolaryngology, Basel University Hospital USB, Basel, Switzerland; 2 Department of Otorhinolaryngology, Lausanne University Hospital CHUV, Lausanne, Switzerland

**Keywords:** subglottic stenosis, airway stent, pediatric

## Abstract

**Objective:**

We report the surgical outcomes and functional results in a pediatric population following the use of the laryngotracheal LT-mold^R^ prosthesis to treat complex glottic and subglottic stenosis.

**Methods:**

A retrospective observational study in children following open and endoscopic surgical treatment for LT stenosis.

**Results:**

Among 46 patients, 91% received LT-mold during an open surgery and 9% had it following an endoscopic procedure. 93% patients were successfully decannulated and 80% needed stent placement for longer than 2 months. Mean time to decannulation was 229 days. Currently, 83% patients have normal breathing, 67% patients have normal voice or mild dysphonia and swallowing outcomes have remained similar in the pre- and post-operative period.

**Conclusion:**

The LT-mold provided an adequate airway stenting, enabling decannulation in most patients with advanced grades of laryngotracheal stenosis. Duration of stenting and time to decannulation showed no correlation with the grade of stenosis or patient comorbidities. Functional results were optimal.

## Introduction

Laryngotracheal stenosis (LTS) can be congenital, acquired, or mixed in origin and can involve both glottic and subglottic regions, either isolated or simultaneously. Management can be performed endoscopically or through open procedures. The two main surgeries for treating subglottic stenosis (SGS) are cricotracheal resection and anastomosis (CTR),^1^ in which the stenosed area is resected, and the distal airway reconnected to the larynx; and laryngotracheal reconstruction (LTR) where cartilage grafts are used to expand the subglottis. An extended CTR (ECTR) combines resection of the pathological airway along with subglottic expansion.

During airway reconstructive surgery, laryngotracheal stenting is sometimes necessary^2^^,^^3^ to support and immobilize the cartilage grafts and mucosal flaps in the reconstructed airway.^4^ This allows proper healing with the vocal cords in an abducted position, creating an optimal inter-arytenoid and posterior glottic space.

However, stents should not be used unless necessary, since they can act as foreign bodies and induce mucosal injuries, ulcerations, granulation tissue formation and subsequent restenosis, particularly if they do not conform perfectly to the inner laryngeal contours or if they are too rigid. Various authors^2^^–^^6^ have reviewed the features of an ideal stent and identified five key characteristics: (a) the stent should be available in various sizes (both in diameter and length); (b) it should not cause any collateral damage to the respiratory tract; (c) it should avoid foreign body reactions, pressure necrosis or discomfort; (d) food intake should be easy and without aspiration; and (e) post-deployment examination and removal should be simple. Currently, several stents such as the custom-made rolled silastic sheet, Aboulker stent,^7^ Montgomery T-tube,^8^ the Eliachar laryngotracheal stents,^9^ and the Rutter suprastomal stent (Boston Medical Products Inc, USA), are used in airway reconstructive surgeries. Comparing these various stents is however challenging due to the lack of uniform protocols in their use and data collection.^10^

The Monnier LT mold^R^ is a silicone stent designed for temporary airway stenting following surgical reconstruction of cicatricial stenoses of the larynx. Made from implant-grade silicone, it is non-reactive, non-irritating and may be left in place for an unrestricted period.^1^^,^^5^^,^^11^ The mold is hollow and custom-shaped following injection of silicon in pediatric and adult cadaver larynges. Following an airway reconstruction, it supports the airway, immobilizes cartilage grafts and mucosal flaps at the recipient site, and maintains the vocal cords in the abducted position during the healing phase, adequately expanding the respiratory posterior glottis. The LT mold is designed to exert minimal mucosal pressure due to its soft silicon material, but also to resist compressive forces, maintaining the LT anatomy, and moving with the larynx during respiration and swallowing, due to its intra-operative fixation. This helps prevent pressure necrosis, mucosal injuries, and granulation tissue formation at the stent-mucosal interface. The stent may be implanted during an open surgery or endoscopy and is removed endoscopically after the appropriate period of stenting has elapsed. It is available in 10 different sizes (from 6mm to 15 mm in diameter)^11^ for use in infants, children, and adults. Additionally, each size exists in four lengths allowing for precise accommodation of the tracheostoma location relative to the glottis. Multiple different stent sizes and width combinations have been manufactured for use after considering the natural laryngotracheal growth and the stoma site at the time of surgery across different pediatric age groups.

In our unit we have used the laryngotracheal LT-Mold^R^ (CHUV, Lausanne, Switzerland) since more than 2 decades in the treatment of pediatric LTS and this report adds up to our units’ previous publications.^5^^,^^11^

## Methods

This was a retrospective observational study conducted between September 2021 and September 2023. Subjects were selected from a retrospectively collected database of patients treated using the LT-mold, operated between January 2010 and December 2020. Seventy-seven patients under the age of 18 were identified, whose indication for surgery was glottic and/or subglottic stenosis. We excluded patients treated for isolated tracheal lesions without glottic, or subglottic involvement, as well as those with stenosis related to malignant conditions or those who received LT-mold placement for chronic aspiration. Finally, our study consisted of 46 patients.

Preoperative evaluation was done as per the European Laryngology Society recommendations.^12^ Laryngeal stenosis was assessed according to grade and location. For congenital glottic webbing we used the Cohen’s classification,^13^ for posterior glottic stenosis the Bogdasarian classification,^14^ and for subglottic stenosis the Cotton-Myer’s classification.^15^ No electromyographic studies were performed. Passive palpation of arytenoid cartilages was done for the diagnosis of cricoarytenoid ankylosis. Selection of patients and use of the LT-mold during endoscopic treatment and open surgery was made as per prior publications.^16^^,^^17^ All procedures were staged, and the tracheostomy was removed at a later date, following a successful decannulation trial.^18^

**Figure 1.**
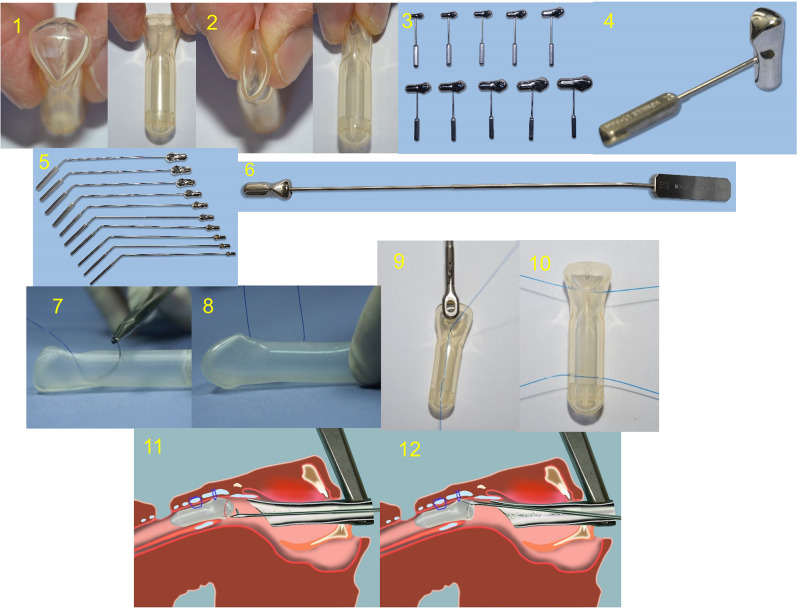
LT-mold and its fixation during an endoscopic or open airway surgery. 1. The top and front view of the LT-mold. Please note the multiple V letter markings on the head of the prosthesis, the acute angle points towards the anterior laryngeal commissure. 2. The LT-mold is hollow and can be pinched easily without losing its form on release. 3, 4. Metal templates for per operative fixation of the LT-mold during an open airway surgery. 5, 6. Metal template for an endoscopic airway fixation of the LT-mold. 7, 8, 9. For an endoscopic fixation of the LT-mold (please see the electronic supplement), a single 3.0 Prolene stitch is passed just below the beak and the two suture limbs fed into the needle of the Lichtenberger carrier. The intralaryngeal mold deployment is done using a heavy forceps. 10. Fixation of the LT-mold during an open surgery is done using two 3.0 Prolene sutures that doubly fixes it in the supraglottis and the trachea. 11. Removal of the LT-mold (electronic supp.) is done under suspension microlaryngoscopy. Using microlaryngeal scissors, a hole is made into the head to expose the Prolene fixation stitch(s). 12. Under telescopic guidance, the fixation stitch(s) are cut, and the LT-mold is removed.

The endoscopic fixation (Fig. 1; *and electronic supplement*) was done under suspension laryngoscopy using the Parsons’ laryngoscope with a side slot (8576 B, Karl Storz, Germany) and a Hopkins rod telescope. The fixation was done using 3.0 Prolene suture (70cm long), that was first passed into the LT-mold outside the patient’s body. The needle entered just below the beak of the stent and exited vertically in the distal part of the LT-mold. The two Prolene arms (glottic and tracheal) were then fed into the needle of a Lichtenberger needle carrier (LNC Ref. 8276.951 Richard Wolf, Germany) and were passed inside-to-outside and caudo-cranially. The LT-mold was then deployed into the larynx using a stout laryngeal forceps (Laryngoforce II 8662 GL, Karl Storz, Germany) and the 2 arms were knotted in the subcutaneous tissue. The ideal position of the mold would have the sharp angle of the beak lying in the anterior laryngeal commissure (ALC), and the distal end being flush at the upper edge of the tracheostoma.

During an open surgery, the LT-mold was fixed using two 3.0-Prolene sutures that were passed horizontally (Fig. 1), fixing it in the *supraglottis* at the level of the false cords and the *suprastomal trachea* at the junction of the distal cap, where the LT-mold wall is the thickest. This significantly diminishes the risk of spontaneous mold extrusion.

To select the correct size of the mold during an open and endoscopic procedure, we used metal templates with laryngeal contours (Fig. 1; show the templates used at open surgery; *5-6* during an endoscopic use). The length was measured from the anterior laryngeal commissure (ALC) up to the upper margin of the stoma. Seven to ten days after the surgery, patients had an endoscopy using 0° (passed trans-orally) and 70° (passed through the tracheostoma) telescopes to see the proximal and distal ends of the LT-mold.

**Figure 2.**
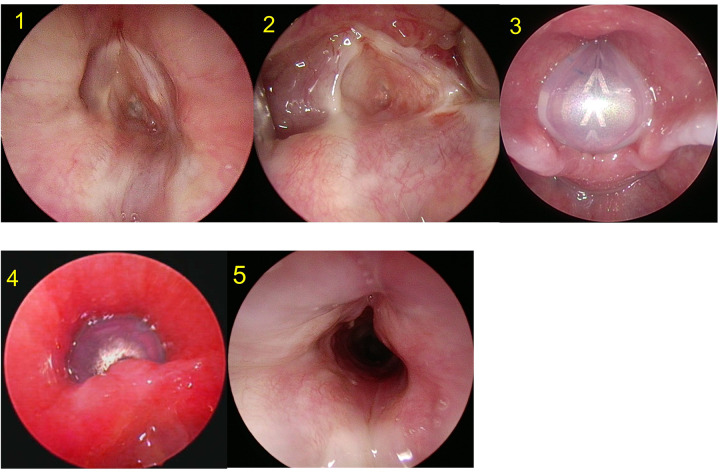
Use of the LT-mold in an open airway surgery. 1, 2. Endoscopic view in a 3-year-old patient with grade IV glotto-subglottic stenosis. 3.Trans-oral endoscopic view of the proximal end of the LT-mold following an extended partial cricotracheal resection and anastomosis (please note that the V mark points towards the anterior laryngeal commissure). 4. Trans-stomal view of the distal end of the LT-mold. 5. Post-operative view. The child was successfully decannulated.

The decision on the duration of keeping the LT-mold in-situ was made by the senior author as per each case and removed under suspension micro-laryngoscopy (SML) (Fig. 1; *electronic supp*, Fig. 2) Use of the LT-mold in an open airway surgery. After LT mold removal, minor granulations were removed using cold-steel biopsy forceps with topical adrenaline application for hemostasis. Major granulations needed removal, and in some cases application of Mitomycin C^19^ and endoscopic re-insertion of the LT-mold. Following LT-mold removal, patients received oral antibiotics, systemic and aerosolized steroids for 5-7 days and proton pump inhibitors for 4-6 weeks.^20^ Patients were decannulated as per published recommendations.^18^ All patients had at least one endoscopy in our unit prior to their discharge after being decannulated. Patients with complex stenosis and multiple past interventions needed longer duration of stenting. In them, the LT-mold was removed at 3-4 months interval under SML and a new LT-mold of similar or larger size was re-inserted endoscopically.

During the stay at our hospital, our pediatric speech and swallow therapists evaluated the voice and the swallowing, and after returning to their countries, the functional results were evaluated by the referring medical team. Voice quality was evaluated as per the parents’ response to questionnaires used in a previous publication^21^ by our unit and was classified as follows: 1. Normal voice. 2. Mild dysphonia (hoarse voice with some difficulties to hear or understand in a noisy environment). 3. Moderate dysphonia (weak voice or ventricular band phonation with easy fatigability). 4. Severe dysphonia (breathy voice with difficulty to communicate). Swallowing was described as: 1. Normal, 2. Mild dysphagia (oral feeding possible but difficult with some textures, and no aspiration), 3. Moderate to severe dysphagia (difficult with all textures with aspiration risk and need to place a nasogastric tube or PEG). The most recent endoscopy findings, respiration, voice, and swallowing were communicated to us by electronic mails sent by the referring doctors.

The study was approved by the institutions Ethics Committee (CER VD 2021-00106), and being a retrospective study, parental consent was waived. The collected information included demographics (sex, age); presence of comorbidities and malformations; respiratory, vocal and swallowing status before and after the surgery; stenosis assessment (etiology, grade, type, location, vocal fold mobility); history of previous treatment; type of surgery performed; decannulation date and time to decannulation; duration of stent placement; complications related to the surgery and to the stent use; and need for revision open surgery. Patient data were documented on an Excel sheet (Microsoft) and analyzed using the SPSS (IBM Corp., Version 28.0) Software.

## Results

### Patient characteristics (Table 1)

Our cohort included 46 patients, of which thirty-one were males (67.4%), and the mean age at operation was 2.9 years (range was 3months, 8days up to 17years, 2months). The mean follow-up time was 33.5 months (range: 3-64 months). Twenty-six (56.5%) patients had a history of prematurity, and 16 were born extremely premature (< 28 weeks). Thirty-one (67.4%) patients had stenosis related to prolonged intubation.^22^ Most of our patients (n=43, 93.4%) were referred from other national and international centers and 22 (47.8%) of them had received previous treatment prior to their referral to our institution; and 5 had multiple (>2) past open airway interventions.

**Table 1.** Patient characteristics

Demographics	
N	46
Age, mean (y) ± SD	2,96 (4,01)
Sex (Male: Female)	31:15
Prematurity (n)	26 (56,5%)
– extreme premature	16 (34,78%)
Associated malformations*	23 (50%)
– Syndromic**	5 (10,86%)
Referred cases, n (%)	43 (93,41%)
Previous treatment	22 (47,83%)
– Endoscopic	14 (30,43%)
– Surgical	8 (17,39%)
Follow-up in days (mean, SD)	514.13 (SD 495)
**Description of the Stenosis**	
Etiology	
– Acquired	27 (58,7%)
– Congenital	13 (28,26%)
– Mixed	6 (13,04%)
Location	
– Subglottic	13 (28,26%)
– Glottic (posterior glottic PGS)	6 (13,04%)
– Glotto-Subglottic	27 (58,7%)
Grade	
1] High grade^+^	34 (73,91%)
– Subglottic	7 (15,22%)
– Glottic	4 (8,7%)
– Glotto-Subglottic	23 (50%)
2] Low grade^++^ 12 (26.09%)	
– Subglottic	6 (50%)
– Glottic 4 (33.3%)	
– Glotto-Subglottic	2 (16.6%)
Vocal Cord mobility	
– Normal	16 (34,78%)
– Hypomobility-immobility	30 (65.22%)
Unilateral CAA	5 (16,66%)
Bilateral CAA	16 (53,33%)

* Associated malformations: patent arterial duct, interventricular communication, duplicate superior vena cava, patent foramen ovale, bicuspid aortic valve, pig bronchus, tracheo-oesophageal fistel, cleft palate, microtia, microcephalie, syndactilie, equin varus feet, pectus scavatum, anal malformation and colon malformation, kidney agenesia, hypospadias.

** Pierre Robin, Stickler syndrome, Frasier syndrome, Down syndrome, Di George syndrome. CAA-cricoarytenoid ankylosis;

+ Cotton-Myer grade III, IV &/or Bogdasarian grade 3, 4

++ Cotton-Myer grade II &/or Bogdasarian grade 2

Thirty-four patients (73%) had severe degree of stenosis (Cotton-Myer grade III, IV and /or grade III/IV posterior glottic stenosis) and 30 patients (65%) had vocal cord(s) immobility.

### Surgical Data (Table 2)

More than 90% insertions of the LT-mold were done during an open surgery (CTR/E-CTR=20, LTR=22). The mean duration of LT-mold stenting (DoS) was 105 days (range: 18-192). The DoS was more than 2 months in 36 patients (83.7%), and 7 patients (16.2%) required the LT-mold for > 6 months. Twenty-one patients (45.6%) with severe comorbidities (extreme prematurity, syndromic) required stenting for up to 2 months.

**Table 2.** Surgery details

Intervention with LT-Mold	
Primary operation^+^	
– CTR/ E-CTR	20 (43.48%)
– LTR	22 (47.83%)
– Endoscopic (PCS & PCCG)	4 (8.69 %)
Duration of LT-Mold use in days (mean, range)	105 (range 18 – 192)
Time to decannulation in days: mean (SD)	228.53 (343)
– 18−60 days	7 (13.04%)
– 61−180 days	29 (54.34%)
– >181 days	7 (16.2%)

+ Double stage procedure. Abbreviations: CTR-cricotracheal resection; E-CTR- Extended CTR; LTR-laryngotracheal reconstruction; PCS-posterior cricoid split; PCCG-posterior costal cartilage graft

Currently, 43 patients (93.4%) are successfully decannulated, with an average time to decannulation (TTD) of 229 days (range: 36 days – 5years, 2months,16days). The three patients who are still dependent on tracheostomy had multi-site severe grade stenosis and are syndromic.

The TTD was longer by 87 days (41.5%) in cases of severe stenosis with an average of 253 days (range: 36-1902 days), versus 166 days (range: 54-824 days) for low-grade stenosis. We performed a Pearson analysis, that showed no correlation between duration of stenting (DoS) and TTD (r = 0,36). Additionally, the Mann Whitney Test showed no statistical difference in the DoS and TTD in patients with- and without comorbidities (*Z* = -0.41; *p* = 0.67), even though patients with comorbidities had longer median DoS (+5 days).

Postoperative complications included moderate (n=9) to severe granulations (n=2) in the glottic-subglottic and suprastomal regions. These eleven patients received endoscopic treatment for the granulations and required an additional period (median:93days; IQR:62-366days) of stent placement. Temporary re-stenting was done endoscopically (LT-mold in 7 patients; Montgomery T-Tube in 4 patients). All these patients had complex multi-site stenoses, and an age-appropriate airway was eventually obtained in 9 of them. Two patients with severe granulations required revision open airway surgery. Minimal granulations were seen in 29 patients and required no further interventions apart from simple cold-steel ablation. One patient had a spontaneous extrusion of the LT-mold and needed its reinsertion. Major complications (granulations and expulsion) were seen in 12 (26%) patients.

### Functional results (Table 3)

Postoperatively, breathing was normal in 38 patients, and 39 patients (85%) had an age-appropriate airway at the last endoscopy. Decannulation was not possible in three patients: in one case the parents have refused further treatment, and in the other two, decannulation has not yet been done, though both patients tolerate full capping.

**Table 3.** Functional outcomes

Functional Results	
**Preoperative Assessment**	
Breathing	
– Tracheotomy	41 (89.13%)
– DoE	5 (10.86%)
Voice	
– Normal	6 (13.04%)
– Slight dysphonia	6 (13.04%)
– Moderate to severe dysphonia	15 (32.6%)
– Aphonia	9 (41.3%)
Swallowing	
– Normal	29 (63%)
– Enteral feeding (NGT/PEG) 17 (37%)	
**Postoperative Assessment**	
Postoperative Breathing (n)	
– Normal	38 (82.6%)
– DoE	5 (10.9%)
– Tracheostomy	3 (6.5%)
Postoperative Voice	
– Normal	18 (39.1%)
– Slight dysphonia	13 (28.3%)
– Moderate to severe Dysphonia	15 (32.6%)
Postoperative Deglutition	
– Normal	32 (69.6%)
– Enteral feeding 14 (30.4%)	
Post-op airway size	
– 90−100%	39 (84.8%)
– 70−89%	5 (10.9%)
– 50−69%	2 (4.3%)

Abbreviations: DoE- Dyspnea on effort; NGT-nasogastric tube; PEG-percutaneous endoscopic gastrostomy

Regarding voice outcomes following the LT-mold use: more than two-third of patients (67.4%, n=31) showed an adequate anterior commissure shape and angle during endoscopy and achieved normal voice or had mild dysphonia. Based on severity of stenosis, voice improvement was seen in 28 (82.4%) of the 34 patients with high-grade stenosis, and in 7 (58.3%) of the 12 patients with low-grade stenosis. Based on the location of the stenosis, 87% (29 out of 33) patients with glottic involvement reported improvement in their voice quality and had better communication as compared to before the surgery. The analysis of voice improvement, or worsening after surgery, showed however no statistical difference in relation to the site (X^2^, *p* = 0,4) or grade of stenosis (X^2^, *p* = 0,5). Swallowing outcomes remained similar in the pre- and post-operative period.

## Discussion

We report here our experience using the Monnier LT-Mold^R^ in pediatric patients undergoing open and endoscopic airway operations. The primary outcome noted was successful decannulation and secondary outcomes were voice and swallowing quality. Majority of the patients (>90%) were referred from external centers and therefore objective evaluation of functional outcomes was sometimes challenging. Our main findings were: 1) airway stenting was adequate and most patients with advanced grades of LTS were decannulated; 2) the duration of stenting did not affect the time to decannulation; 3) subjective voice results were satisfying after LT-mold stenting.

Until the writing of this submission, the LT-mold has been used in 148 pediatric patients with glotto-subglottic stenosis in our unit and this report adds up to the previous publications.^23^^–^^25^ We highlight below our experience while using the LT-mold:

### Selection of LT-mold size and length

During LTR and CTR, the distance from the anterior laryngeal commissure (ALC) to the upper margin of the tracheostoma is recorded. While the airway is still open, and the posterior airway reconstruction has been completed, the LT-mold is selected and tested for proper diameter and length. Adequate and non-tight closure of the thyroid alae over the LT-mold at the level of the ALC indicates that the prosthesis is of an appropriate size. Further need for an anterior subglottic enlargement with a costal cartilage graft can be assessed while the LT-mold is in place.

Selecting the correct size and length of the LT-mold is critical to ensure optimal airway results and avoid complications,^2^ and for this we use pre-formed metal templates having an endo-laryngeal shape. A *too-large* LT-mold may cause decubitus lesions in the newly reconstructed airway, causing an anastomotic dehiscence and/or loss of the expansion cartilage graft. A *too-small* size will not sufficiently stent the airway, cause recurrence of stenosis, and will leave a gap posterior to the mold causing saliva/food to leak distally and cause infection at the reconstruction site and in the lungs. This distal leakage can be temporarily treated by injecting hyaluronic acid fillers in the posterior glottis. A *too-long* LT-mold can block the stoma making cannula changes difficult and a mold *too-short* may not effectively prevent suprastomal granulations from forming.

### Stent complications

The LT-mold is soft, has smooth edges cranially and caudally, thus avoiding further ulceration and granulation tissue formation at this level. It is fixed to the trachea and the thyroid cartilage to avoid migration. As the mold’s head is larger than the tracheal diameter, distal dislodgement into the lower trachea and bronchi is impossible even during tracheostomy cannula cleansing and replacement. The LT-mold can, however, be expelled through the mouth or be inadvertently swallowed, but being oval-shaped and made of soft silicone, it progresses through the digestive tract and is eliminated in the feces. One of our patients tended to frequently handle the cannula that pushed the mold cranially, caused loosening of the anchoring Prolene stitches and eventually caused its expulsion.

We saw moderate to severe granulations in 11 patients, and they needed an extended period of stent re-insertion. No cases of long-term dysphagia requiring G-tube insertion were noted. The distal round-shaped cap of the LT-mold supports the suprastomal area, minimizes granulation tissue formation, and could potentially prevent suprastomal collapse if it is properly fixed in place. This is the focus of an ongoing study in our unit.

In comparison to the LT-mold, the Aboulker stent is made of polished Teflon, and Zalzal et al.^26^^,^^27^ noted that the hard biomaterial caused severe granulations at the inferior or superior end of the stent, applied pressure necrosis at the base of the epiglottis and medial aspect of the arytenoids causing stent migration and breaking. Furthermore, this cigar-shaped stent cannot restore a sharp angle of the anterior laryngeal commissure in cases of cicatricial fusion of the vocal cords, having a negative impact on the voice quality.

The Montgomery T-tube (MTT)^8^ is soft and pliable, allowing easy insertion through the tracheostoma. While an appropriately sized tube is generally well tolerated by the mucosa, both extremities have sharp cut edges that can promote granulation tissue formation at the site of the shearing forces between the stent and the airway mucosa.^27^^,^^28^ In children, safety concerns exist when MTT less than 8 mm in outer diameter is used, because the tube can get blocked with dry secretions requiring immediate removal.^29^

The Eliachar LT-stent is a hollow stent made of soft silicone with conformity to the inner laryngeal contours, but its shape is not triangular at the level of the glottis.^9^ Additionally, it exists only in two adult sizes and is not adequate for use in infants and children.

Metallic stents^30^ are not being routinely used for laryngeal stenting anymore.

A soft endotracheal tube (ETT) is routinely used as a stent in single stage procedures (LTR and CTR), and its stenting period is limited to 5-7 days.^31^ In double stage procedures, an age-appropriate soft ivory portex ETT can be cut to the desired length and the proximal and distal ends closed using non-resorbable sutures. This custom-made stent^32^ is held in place by a Prolene transfixion suture and removed endoscopically on an average 4-6 weeks later.

Setlur et al.^33^ described the hybrid (*one and half – 1.5*) reconstruction technique which is a combination of single- and double- stage laryngotracheal reconstruction (LTR). The authors used a nasotracheal ETT to function like a stent for the cartilage graft(s) expansion, while a 3.0 neonatal cuffless tracheostomy was placed through the tracheostoma to keep it open, and rested above the cuff of the endotracheal tube. The advantage of this 1.5 technique is to allow passing the tracheostomy cannula into the stoma in the event of a decannulation failure, and the same group^31^ report this technique exclusively in patients undergoing an LTR. They limit the period of stenting with the ETT to less than a week in single stage and 1.5 technique and to 3 weeks in a double stage LTR.

### Duration of stenting

Our results showed no correlation between the time to decannulation and the duration of stenting, the grade of stenosis or patient comorbidities. The decision of long-term stenting was made as per each individual case, relying on our experience with the LT-mold’s tissue-friendliness. This is an advantage in complex, multilevel and multi-operated cases^2^ where long duration of stenting can favor better cicatrization and scar modelling.^34^ We kept the LT-mold for a longer time in children with syndromes and prematurity with an objective of giving additional time for their swallow therapy, and the LT-mold providing security against aspiration. In this way, it is possible that complex patients with LTS could be better prepared for a safe swallowing following their LT-molds’ eventual removal.

### Functional results

In this study, decannulation rates were similar to reports from other experienced centers.^,^^32^^,^^35^^,^^36^ The LT-mold has a unique shape and is ideal to re-establish the inner contours of the larynx, and more importantly, the acute angle of the anterior laryngeal commissure (ALC). More than two-thirds of our patients had an adequate ALC angle post-operatively, and therefore had better voice results. George et al.^18^ concluded that children with associated glottic involvement are at high risk for poor voice outcome following PCTR, though in our cohort, analysis of voice after surgery showed no statistical difference for location or grade of stenosis.

Limitations to our study are its retrospective design. The LT-mold use in glotto-subglottic stenosis is unique to our institution and therefore there was no control group. The patients had different types and grades of stenoses, were operated with different surgical techniques, and therefore evaluation of functional outcomes could be biased. Additionally, the patient’s evaluation could have had inter-observer errors, as significant number of patients continued their rehabilitation at their referring institution. Voice analysis was limited by the usual biases, such as subjectivity of the patients’ or parent’s estimation of the voice, lack of independent evaluation of the voice by multiple observers, and no objective assessment. The referral pattern of our patients is challenging, and efforts are being made with the referring institutions to analyze them objectively. A prospective study comparing the LT-mold with other stents is recommended.

- During airway reconstructive surgery, laryngotracheal stenting is sometimes necessary to support and immobilize the cartilage grafts and mucosal flaps in the reconstructed airway. An ideal airway stent should optimally scaffold the endo-larynx and at the same time avoid collateral damages.
- The laryngotracheal (LT)-mold is an excellent adjunctive device in surgeries for complex glotto-subglottic stenoses that require long-term stenting. It is well tolerated, and patients have excellent optimal postoperative functional results.
- The study showed no correlation between the duration of stenting and time to decannulation with the grade of stenosis and patient comorbidities.

## Conclusion

Laryngeal stents are important adjunctive devices in surgeries for laryngotracheal stenosis. We report that the LT-mold helps solve complex glotto-subglottic stenoses that require long-term stenting, is well tolerated, and patients have satisfying postoperative functional results. The study showed no correlation between the duration of stenting and time to decannulation with the grade of stenosis and patient comorbidities.

## Supporting information

Varela and Sandu supplementary material Varela and Sandu supplementary material

## Supplementary material

The supplementary material for this article can be found at https://doi.org/10.1017/S0022215125102533.

## Electronic supplement: Endoscopic insertion and removal of the LT Mold

*Insertion*: An age-appropriate LT mold is fixed under suspension microlaryngoscopy (SML) using 3.0 Prolene and the Lichtenberger needle carrier.

*Removal of the LT mold that was fixed during an open surgery*: Note the inverted V mark on the head of the prosthesis facing the anterior laryngeal commissure. On touching the hollow and transparent prosthesis, we can see the vocal cords and the transversely placed Prolene sutures. First using microlaryngeal scissors, the head of the LT mold is uncapped to see the Prolene sutures that are then cut, and the mold is removed. The Prolene sutures are retrieved using microlaryngeal forceps. Note the vocal cords mobility and the optimal airway created. The patient was decannulated successfully.
